# Circulating tumour DNA for monitoring colorectal cancer—a prospective cohort study to assess relationship to tissue methylation, cancer characteristics and surgical resection

**DOI:** 10.1186/s13148-018-0500-5

**Published:** 2018-05-16

**Authors:** Erin L. Symonds, Susanne K. Pedersen, David H. Murray, Maher Jedi, Susan E. Byrne, Philippa Rabbitt, Rohan T. Baker, Dawn Bastin, Graeme P. Young

**Affiliations:** 1 Flinders Centre for Innovation in Cancer, College of Medicine and Public Health, Flinders University of South Australia, Bedford Park, South Australia 5042 Australia; 2 Bowel Health Service, Flinders Medical Centre, Bedford Park, South Australia Australia; 3 Clinical Genomics Pty Ltd, North Ryde, New South Wales Australia; 4 Colorectal Surgery, Division of Surgery and Perioperative Medicine, Flinders Medical Centre, Bedford Park, South Australia Australia

**Keywords:** Colorectal cancer, *BCAT1*, *IKZF1*, Methylation, Circulating tumour DNA, Surgical resection, Residual disease

## Abstract

**Background:**

Cell-free circulating tumour-derived DNA (ctDNA) can be detected by testing for methylated *BCAT1* and *IKZF1* DNA, which has proven sensitivity for colorectal cancer (CRC). A prospective correlative biomarker study between presence of methylated *BCAT1* and *IKZF1* in tissue and blood was conducted in cases with CRC to explore how detection of such ctDNA biomarkers relates to cancer characteristics, methylation in tissue and surgical resection of the primary cancer.

**Methods:**

Enrolled patients with invasive CRC had blood collected at diagnosis, prior to any treatment or surgery (peri-diagnostic sample). A subgroup of patients also had cancer and adjacent non-neoplastic tissue collected at surgical resection, as well as a second blood sample collected within 12 months of surgery (post-surgery sample). DNA was extracted from all samples and assayed for methylated *BCAT1* and *IKZF1* to determine the degree of methylation in tissue and the presence of ctDNA in blood.

**Results:**

Of 187 cases providing peri-diagnostic blood samples, tissue was available in 91, and 93 provided at least one post-surgery blood sample for marker analysis. Significant methylation of either *BCAT1* or *IKZF1* was seen in 86/91 (94.5%) cancer tissues, with levels independent of stage and higher than that observed in adjacent non-neoplastic specimens (*P* < 0.001). ctDNA methylated in *BCAT1* or *IKZF1* was detected in 116 (62.0%) cases at diagnosis and was significantly more likely to be detected with later stage (*P* < 0.001) and distal tumour location (*P* = 0.004). Of the 91 patients who provided pre-and post-surgery blood samples, 47 patients were ctDNA-positive at diagnosis and 35 (74.5%) became negative after tumour resection.

**Conclusion:**

This study has shown that *BCAT1* and *IKZF1* methylation are common events in CRC with almost all cancer tissues showing significant levels of methylation in the two genes. The presence of ctDNA in blood is stage-related and show rapid reversion to negative following surgical resection. Monitoring methylated *BCAT1* and *IKZF1* levels could therefore inform adequacy of surgical resection.

**Trial registration:**

Australian New Zealand Clinical Trial Registry number 12611000318987. Registered 25 March 2011.

**Electronic supplementary material:**

The online version of this article (10.1186/s13148-018-0500-5) contains supplementary material, which is available to authorized users.

## Background

Colorectal cancer (CRC) is the second leading cause of death from cancer in the developed world [1]. Even though most CRC patients achieve remission with initial treatment, more than 25% will suffer recurrence [2]. Therefore, to achieve early detection of recurrence, patients are usually entered into a follow-up regimen including regular blood testing, radiology and colonoscopy. The current blood biomarker used, carcinoembryonic antigen (CEA), has limited sensitivity and specificity for recurrence [3, 4]. Better blood markers for adequacy of initial therapy should aid identification of subjects at risk of recurrence or in need of extended initial therapy such as addition of or prolongation of chemotherapy. These markers should be present in all colorectal tumours regardless of the genetic alterations and should be absent from the blood following complete surgical resection of the tumour.

Somatic and epigenetic alterations of DNA are associated with CRC development, and a number of studies show that tumour-derived DNA can be detected in the cell-free fraction of blood (circulating tumour DNA, ctDNA) [5–8]. Detecting ctDNA by assaying for somatic mutations [9, 10] has the potential to inform response to therapy, existence of minimal residual disease and development of metastases [11, 12]. However, none of the common somatic mutations linked to CRC development occurs universally, and all appear with a low frequency [5, 13]. Furthermore, the mutation profile becomes more heterogeneous as the cancer evolves over time, whereas epigenetic markers are more stable during oncogenesis [5]. Thus, aberrant DNA methylation, which occurs frequently in CRC [5, 6] might more reliably inform response to therapy and presence of residual disease.

A panel of methylated DNA biomarkers shown to have good sensitivity and specificity for CRC is *BCAT1* (branched chain amino acid transaminase 1) and *IKZF1* (IKAROS family zinc finger 1) [14–16]. Deregulation of *IKZF1* and *BCAT1* is involved in tumour growth and invasiveness in several cancers, including CRC [6, 17]. There is a biological plausibility that methylated *BCAT1* and *IKZF1* may be more significant in oncogenesis than simply epiphenomena resulting from disturbances in gene methylation processes as both play an important functional role in maintaining a healthy state in normal tissue [18–20]. BCAT1 controls the metabolism of branched chain amino acids which are essential nutrients for growth, and it has been demonstrated that when *BCAT1* expression is blocked, the lifespan increases nearly 25% in nematodes [21]. The *BCAT1* gene locus is aberrantly methylated in several pathologies, including CRC [20, 22] where abnormal expression has been reported to be a predictor of distant metastases [17]. *IKZF1* encodes a DNA-binding protein, which during normal development restricts the G1-S transitioning of the cell cycle by regulating a small set of cell cycle regulator genes [23, 24]. *IKZF1* mutations and deletions leading to generation of isoforms lacking DNA binding capability are common in hematologic neoplasia, e.g. lymphoblastic leukemia where such mutations/deletions abolish cell cycle control and leads to hyperproliferation. In CRC, *IKZF1* promoter methylation has been linked to loss of proper regulation of proliferation and differentiation [24].

If detection of methylated *BCAT1*/*IKZF1* ctDNA is to be useful in patient management, it is important to better understand the principles underlying the presence of these epigenetic markers in blood and how this relates to tissue expression and to tumour debulking. The aim of this study was therefore to assess the relationship between tissue levels and detection in blood and to examine the effects of surgical resection on presence of ctDNA.

## Methods

### Study overview

This was a prospective observational study of cases with invasive CRC that examined the relationship of ctDNA status with clinicopathological measures and with levels of methylated *BCAT1* and *IKZF1* DNA in surgically resected tissues. In addition, the effect of surgical resection on ctDNA status was assessed. A methylation-specific validated real-time PCR-based method was used to assess the presence of methylated *BCAT1* and *IKZF1* in bisulphite-converted DNA isolated from plasma [25]. The degree of methylation of *BCAT1* and *IKZF1* DNA were also measured in tissue (tumour and adjacent non-neoplastic epithelium).

The study was approved by the Southern Adelaide Clinical Human Research Ethics Committee (reference number 134.045). Written informed consent was obtained from all participants. The study is registered at Australian and New Zealand Clinical Trials Registry (#12611000318987).

### Population

Any adults (18 years of age or older) who were recently diagnosed with invasive colorectal adenocarcinoma (AJCC stages I–IV) at Flinders Medical Centre (Bedford Park, SA, Australia) or Repatriation General Hospital (Daw Park, SA) were approached about volunteering for the study during 2011–2016. Following consent, patients were enrolled in the study provided that they met diagnostic criteria for invasive colorectal adenocarcinoma, were adequately staged, and were provided a blood sample prior to any treatment (the peri-diagnostic sample). Diagnosis and extent of disease were determined on the basis of colonoscopy and other clinicopathological findings. CRC were staged (following AJCC and TNM) [26], and distal tumours were classed as those distal to the transverse colon.

### Clinical procedures

Venous blood for ctDNA testing was collected into two 9 mL K3EDTA Vacuette tubes (Greiner Bio-One, Frickenhausen, Germany) prior to any treatment including primary surgery from 187 participants (“peri-diagnostic plasma sample”) and when feasible at subsequent clinical review within 12 months after surgery from 93 participants (“post-surgery plasma sample”). Blood collection tubes were kept on ice prior to plasma processing (no more than 4 h from blood collection). Plasma was prepared by centrifugation at 1500*g* for 10 min at 4 °C (deceleration at lowest setting), followed by retrieval of the plasma fraction and a repeat centrifugation. The resulting plasma was stored at − 80 °C. Frozen plasma samples were shipped on dry ice to Clinical Genomics (North Ryde, NSW, Australia) and stored at − 80 °C until testing.

Resected tissue samples were also available for a subgroup of patients who had received no neoadjuvant therapy (*n* = 91). Samples collected were fresh (non-fixed) non-necrotic cancer tissue and adjacent non-neoplastic tissue (greater than 10 mm from the tumour, median 75 mm) which were obtained by a supervising pathologist. Samples were stored in RNAlater (Thermo Fisher Scientific Australia) for at least 48 h at 4 °C before stored at − 80 °C until further analysis.

No study-wide control of radiological imaging, pathology procedures, or quality was undertaken as the study aimed to assess marker performance relative to outcomes determined in usual clinical practice. All procedures were performed by hospital-accredited specialists and so met site-specific standards for venipuncture, monitoring, imaging and equipment.

### Methylation testing

For ctDNA testing, cell-free circulating DNA was extracted from 4.5 mL plasma using the QIASymphony Circulating Nucleic Acid Kit (Qiagen, Hilden, Germany) and bisulphite-converted using the EpiTect Fast Bisulphite Conversion kit (Qiagen) as previously described [16]. The resulting bisulphite-converted DNA was simultaneously analyzed in triplicate using a real-time multiplex PCR assay simultaneously detecting a methylated region in *BCAT1* and *IKZF1* as well as a region in *ACTB* (proxy measure of the total amount of DNA) using a LightCycler 480 II instrument (Roche Diagnostics, IN, USA) [16, 25, 27]. We have previously shown this assay to be sensitive for the detection of low copy numbers of the methylated genes [25]. A blood sample was deemed ctDNA positive for clinical purposes if at least one PCR replicate was positive for methylated *BCAT1* and/or *IKZF1* [16, 27].

For tissue DNA analysis, DNA was extracted from 10 to 20 mg tissue according to the manufacturer’s instructions (DNAeasy® Blood & Tissue kit, Qiagen), except for using 40 μL Proteinase K and a lysis time of 3 h at 56 °C. DNA (500 ng) was bisulphite-converted using an EpiTect Fast 96 Bisulphite Conversion kit (Qiagen) as previously described [16] with the exception of omitting carrier RNA and a 30 μL elution.

When consideration was given to percentage of methylated ctDNA, the level was expressed as the ratio of total mass of *BCAT1* and *IKZF1* measured in total amount of DNA volume. A sample was deemed positive when the %methylation was above the 75th percentile value of *BCAT1* and *IKZF1* of the non-neoplastic tissue (9.7% for *BCAT1* and 0.5% for *IKZF1*).

### Statistical analyses

Hypothesis testing included Mann-Whitney, Kruskal-Wallis, chi-square and Fisher’s exact tests. Analyses were performed using two-sided tests, and a significance level of less than 5% was considered statistically significant. The binomial distribution was assumed for calculations of exact 95% confidence intervals (95%CI). All analyses were performed using Stata, version 13.1.

Once the data was collected, a power analysis was performed for a logistic regression analysis to compare the blood positivity across different cancer characteristics. The analysis was performed using a *z* test with a binomial distribution, an alpha of 5%, plasma positivity of 62% and a proportion comparison of 0.5 to 0.7. A power of 0.78 was estimated.

Logistic regression was used for multivariate analysis. Variables that were included were those where the relationship with the outcome was significant at *P* ≤ 0.1 in the univariate analysis or had been shown in previous studies to be clinically significant. Age, tumour size, lymphatic invasion, perineural invasion, extramural vascular invasion, intramural vascular invasion and differentiation were included in the multivariate analysis. The final model was prepared using a backward selection method, and the goodness of fit was assessed using the Pearson chi-square test.

## Results

### Study population

**Fig. 1:**
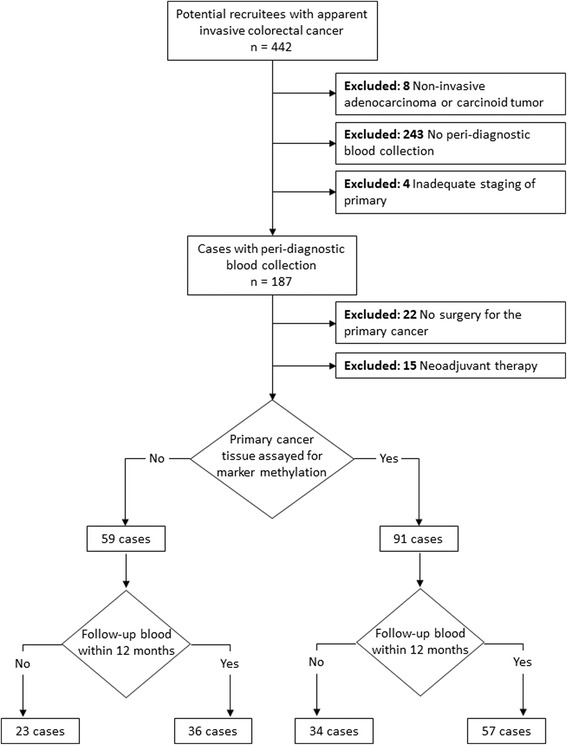
Disposition of study cohort. Peri-diagnostic blood collection refers to sampling either prior to diagnostic procedure or between that and surgery

Figure 1 shows the disposition of 442 patients approached. The characteristics of cases according to clinical information and specimen availability are summarized in Additional file 1: Table S1. The stage distribution differed between all cases and just those cases providing a post-surgical blood sample as not all stage IV cases proceeded to surgery.

### Peri-diagnostic ctDNA status

Peri-diagnostic blood samples were available for 187 cases, and 116 (62.0%, 95%CI 54.7–69.0) tested positive for methylated *BCAT1* and/or *IKZF1*. When methylation changes were assessed for different clinicopathological features, similar results were seen for *BCAT1* and *IKZF1* except for location where *BCAT1* had a higher positivity rate with distally located tumours (Table 1). Overall ctDNA positivity varied by AJCC and TNM staging, size, location and lymphatic invasion by univariate analysis (Table 1). CRC stages II, III and IV were strong predictors for ctDNA positivity compared to stage I. After multivariate modeling, only stage and location remained as significant predictors; patients with tumours located in the distal colon or rectum were 3.0 times more likely to be ctDNA positive than those with proximal tumours (95% CI 1.4–6.2) (Table 2).

**Table 1:** Plasma *BCAT1*, *IKZK1* and overall ctDNA positivity for different clinicopathological findings of patients with invasive colorectal cancer (*n* = 187)

Factor	Category	*N*	Methylated *BCAT1* DNA			Methylated *IKZF1* DNA			ctDNA positivity (combined *BCAT1/IKZF1*)		
			No. positive	% positive (95% CI^1^)	*P* ^2^	No. positive	% positive (95% CI^1^)	*P* ^2^	No. positive	% positive (95% CI^1^)	*P* ^2^
Age	< 65 years	67	29	43.3 (31.2–56.0)	0.154	28	41.8 (29.8–54.5)	0.332	39	58.2 (45.5–70.2)	0.421
	≥ 65 years	120	65	54.2 (44.8–63.3)		59	49.2 (39.9–58.4)		77	64.2 (54.9–72.7)	
Gender	Female	75	34	45.3 (33.8–57.3)	0.270	31	41.3 (30.1–53.3)	0.244	42	56.0 (44.1–67.5)	0.164
	Male	112	60	53.6 (43.9–63.0)		56	50.0 (40.4–59.6)		74	66.1 (56.5–74.7)	
Stage	I	40	4	10.0 (2.8–23.7)	< 0.001	2	5.0 (0.6–16.9)	< 0.001	6	15.0 (5.7–29.8)	< 0.001
	II	54	29	53.7 (39.6–67.4)		25	46.3 (32.6–60.4)		35	64.8 (50.6–77.3)	
	III	63	37	58.7 (45.6–71.0)		36	57.1 (44.0–69.5)		47	74.6 (62.1–84.7)	
	IV	30	24	80.0 (61.4–92.3)		24	80.0 (61.4–92.3)		28	93.3 (77.9–99.2)	
T stage	T1	26	2	7.7 (0.9–22.1)	< 0.001	1	3.8 (0.1–19.6)	< 0.001	2	7.7 (0.9–25.1)	< 0.001
	T2	23	6	26.1 (10.2–48.4)		3	13.0 (2.8–33.6)		8	34.8 (16.4–57.3)	
	T3	96	55	57.3 (46.8–67.3)		56	58.3 (47.8–68.3)		69	71.9 (61.8–80.6)	
	T4	34	24	70.6 (52.5–84.9)		21	61.8 (43.6–77.8)		29	85.3 (68.9–95.0)	
	Unknown	8	7	87.5 (47.3–99.7)		6	75.0 (34.9–96.8)		8	100.0 (63.1–100.0)	
N stage	N0	100	41	41.0 (31.3–51.3)	0.024	36	36.0 (26.6–46.2)	0.007	50	50.0 (39.8–60.2)	0.001
	N1/N2	73	44	60.3 (48.1–71.5)		44	60.3 (48.1–71.5)		56	76.7 (65.4–85.8)	
	Unknown	14	9	64.3 (35.1–87.2)		7	50.0 (23.0–77.0)		10	71.4 (41.9–91.6)	
M stage	M0	147	66	44.9 (36.7–53.3)	0.002	58	39.5 (31.5–47.8)	< 0.001	82	55.8 (47.4–64.0)	0.001
	M1	30	24	80.0 (61.4–92.3)		24	80.0 (61.4–92.3)		28	93.3 (77.9–99.2)	
	Unknown	10	4	40.0 (12.2–73.8)		5	50.0 (18.7–81.3)		6	60.0 (26.2–87.8)	
Size (mm)	< 20 mm	14	2	14.3 (1.8–42.8)	< 0.001	2	14.3 (1.8–42.8)	< 0.001	3	21.4 (4.7–50.8)	< 0.001
	20–50 mm	100	44	44.0 (34.1–54.3)		37	37.0 (27.6–47.2)		56	56.0 (45.7–65.9)	
	> 50 mm	65	44	67.7 (54.9–78.8)		45	69.2 (56.6–80.1)		52	80.0 (68.2–88.9)	
	Unknown	8	4	50.0 (15.7–84.3)		3	37.5 (8.5–75.5)		5	62.5 (24.5–91.5)	
Location	Proximal	75	28	37.3 (26.4–49.3)	0.011	31	41.3 (30.1–53.3)	0.306	37	49.3 (37.6–61.1)	0.011
	Distal	111	65	58.6 (48.8–67.8)		56	50.5 (40.8–60.1)		78	70.3 (60.9–78.6)	
	Unknown	1	1	100.0 (2.5–100.0)		0	0.0 (0.0–97.5)		1		
Lymphatic invasion	Yes	38	24	63.2 (46.0–78.2)	0.014	25	65.8 (48.6–80.4)	0.001	30	78.9 (62.7–90.4)	0.002
	No	121	49	40.5 (31.7–49.8)		44	36.4 (27.8–45.6)		61	50.4 (41.2–59.6)	
Perinueural invasion	Yes	20	9	45.0 (23.1–68.5)	0.860	10	50.0 (27.2–72.8)	0.541	15	75.0 (50.9–91.3)	0.104
	No	138	65	47.1 (38.6–55.8)		59	42.8 (34.4–51.4)		77	55.8 (47.1–64.2)	
Extramural vascular invasion	Yes	9	6	66.7 (29.9–92.5)	0.313	7	77.8 (40.0–97.2)	0.054	8	88.9 (51.8–99.7)	0.089
	No	178	88	49.4 (41.9–57.0)		80	44.9 (37.5–52.6)		108	60.7 (53.1–67.9)	
Intramural vascular invasion	Yes	3	2	66.7 (9.4–99.2)	0.567	2	66.7 (9.4–99.2)	0.481	2	66.7 (9.4–99.2)	0.868
	No	184	92	50.0 (42.6–57.4)		85	46.2 (38.8–53.7)		114	62.0 (54.5–69.0)	
Differentiation	Poor	34	21	61.8 (43.6–77.8)	0.241	20	58.8 (40.7–75.4)	0.124	25	73.5 (55.6–87.1)	0.160
	Moderate	118	54	45.8 (36.6–55.2)		48	40.7 (31.7–50.1)		67	56.8 (47.3–65.9)	
	Well	14	6	42.9 (17.7–71.1)		6	42.9 (17.7–71.1)		7	50.0 (23.0–77.0)	
	Unknown	21	13	61.9 (38.4–81.9)		13	61.9 (38.4–81.9)		17	81.0 (58.1–94.6)	

^1^Confidence interval

^2^*P* value, Pearson’s chi-square test

**Table 2:** Association between tumour characteristics and ctDNA positivity

Factor	Odds ratio (95% CI)	*P* value
Stage		< 0.001
I	1 (reference)	
II	12.5 (4.3–36.6)	
III	16.6 (5.7–48)	
IV	92.1 (16.5–513.8)	
Location		0.004
Proximal colon	1 (reference)	
Distal colon and rectum	3.0 (1.4–6.2)	

### Methylation in colonic tissue

**Fig. 2:**
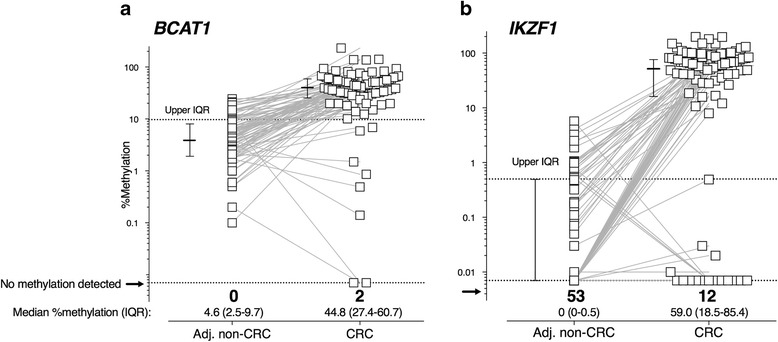
*BCAT1* and *IKZF1* methylation in cancer and adjacent non-neoplastic tissues. Matching data points are indicated by connecting lines. “%methylation”—level of methylated *BCAT1* (**a**) or *IKZF1* (**b**) in 5 ng bisulphite-converted tissue DNA. “Adj. non-CRC”—adjacent non-neoplastic tissue. Cross hairs—median %methylation (horizontal line) and interquartile range (IQR, vertical line), with upper IQR for non-neoplastic tissue represented by the horizontal dotted line

Tumour tissue was available in 91 cases, with matched non-neoplastic tissue in 87. Cancer tissues exhibited significantly greater methylation than adjacent non-neoplastic tissues (*P* < 0.001 for each marker; Fig. 2). Detectable methylation in one or both genes was present in 98.9% (90/91) of cancer tissue, with methylated *BCAT1* present in 89/91 (97.8%) and methylated *IKZF1* present in 79/91 (86.8%). The one tumour that was negative for both *BCAT1* and *IKZF1* methylation had a single somatic mutation in MSH2 and MSH6. Using the upper IQR values measured in non-neoplastic tissues as positivity thresholds, hypermethylation of *BCAT1* and *IKZF1* was observed in 82/91 (90.1%) and 75/91 (82.4%) of cancers, respectively, with 86/91 (94.5%) having elevated levels for either marker. The only variables having any effect on methylation levels in cancers were age older than 65 years and tumour location for *BCAT1* (Table 3). These differences were not seen in non-neoplastic tissue (*P* > 0.05, data not shown).

**Table 3:** *BCAT1* and *IKZF1* methylation in 91 cancer tissue samples according to case demographics and tumour characteristics

			%Methylation, median (IQR)			
Variable factor	Category	*N*	*BCAT1*	*P*	*IKZF1*	*P*
Age	< 65	32	35.8 (14–57.5)	0.039^1^	52.5 (11.4–69.7)	0.143^1^
	≥ 65	59	50.1 (31.4–67.8)		64.8 (31.3–96.3)	
Gender	Female	45	46.9 (31.0–60.7)	0.444^1^	59 (41.6–96.3)	0.359^1^
	Male	46	42.2 (26.9–63.1)		59.3 (10.7–83.3)	
Stage	I	19	50.1 (19.8–81.3)	0.581^2^	56.9 (9.1–115.6)	0.659^2^
	II	34	44.6 (20.2–58.9)		48.7 (0.5–82)	
	III	29	43.3 (32.9–51.9)		61.1 (42.6–84.7)	
	IV	9	57.7 (36.1–71.4)		54.3 (41.4–98.7)	
T stage	T1	8	68.6 (54.4–93.0)	0.066^2^	88.7 (34.4–132.4)	0.362^2^
	T2	13	27.5 (18.8–51.4)		32.1 (9.1–100.3)	
	T3	48	50.5 (32.7–60.0)		65.1 (31.8–84.0)	
	T4	22	32.6 (23.6–52.9)		42.1 (10.7–69.0)	
N stage	N0	57	50.9 (26.9–63.3)	0.680^2^	63.4 (15.8–97.8)	0.538^2^
	N1/N2	33	43.3 (30.3–59.4)		59 (41.4–76.1)	
	Nx	1	26.9 (26.9–26.9)		9.1 (9.1–9.1)	
M stage	M0	79	44.8 (26.9–63.1)	0.282^2^	60.8 (18.5–91)	0.785^2^
	M1	9	57.7 (36.1–71.4)		54.3 (41.4–98.7)	
	Mx	3	31.7 (18.8–35.4)		50.3 (0.0–85.5)	
Size (mm)	< 20 mm	4	59.6 (34.9–103.7)	0.551^2^	68.5 (14.3–152.9)	0.856^2^
	20–50 mm	52	41.5 (27.9–57)		57 (16.2–88.3)	
	> 50 mm	34	47.8 (23.6–67.8)		62.1 (28–84.7)	
	Unknown	1	81.3 (81.3–81.3)		68.8 (68.8–68.8)	
Location	Proximal	50	51.6 (31.0–67.3)	0.029^1^	68.9 (41.6–98.7)	0.055^1^
	Distal	41	37.9 (19.8–51.9)		41.4 (16.6–68.8)	
Lymphatic invasion	Yes	25	42.2 (33.9–63.1)	0.650^1^	63.4 (40.8–85.5)	0.827^1^
	No	66	48.3 (26.9–58.9)		57 (18.5–91)	
Perineural invasion	Yes	13	37.9 (19.9–56.4)	0.626^1^	41.4 (10.7–84.7)	0.708^1^
	No	78	48.3 (27.4–63.1)		59.9 (28.5–96.1)	
Extramural vascular invasion	Yes	4	42.0 (32.2–67.2)	0.816^1^	56.8 (24.6–102.3)	0.816^1^
	No	87	44.8 (26.9–63.1)		59.0 (18.5–91.0)	
Intramural vascular invasion	Yes	1	28.3 (28.3–28.3)	0.446^1^	41.2 (41.2–41.2)	0.594^1^
	No	90	45.8 (26.9–63.1)		59.9 (18.5–91.0)	
Differentiation	Poor	26	47.4 (30.3–67.8)	0.320^2^	59.9 (32.6–96.3)	0.691^2^
	Moderate	53	44.8 (26.9–58.9)		57.1 (12.0–83.3)	
	Well	7	36.1 (19.8–39)		48.7 (18.5–70.6)	
	Unknown	5	56.2 (51.4–63.3)		81.2 (76.3–84.7)	

^1^Mann-Whitney test on medians (two-tailed)

^2^Kruskal-Wallis test on medians (two-tailed)

### Comparison of tumour tissue methylation and peri-diagnostic ctDNA status

**Fig. 3:**
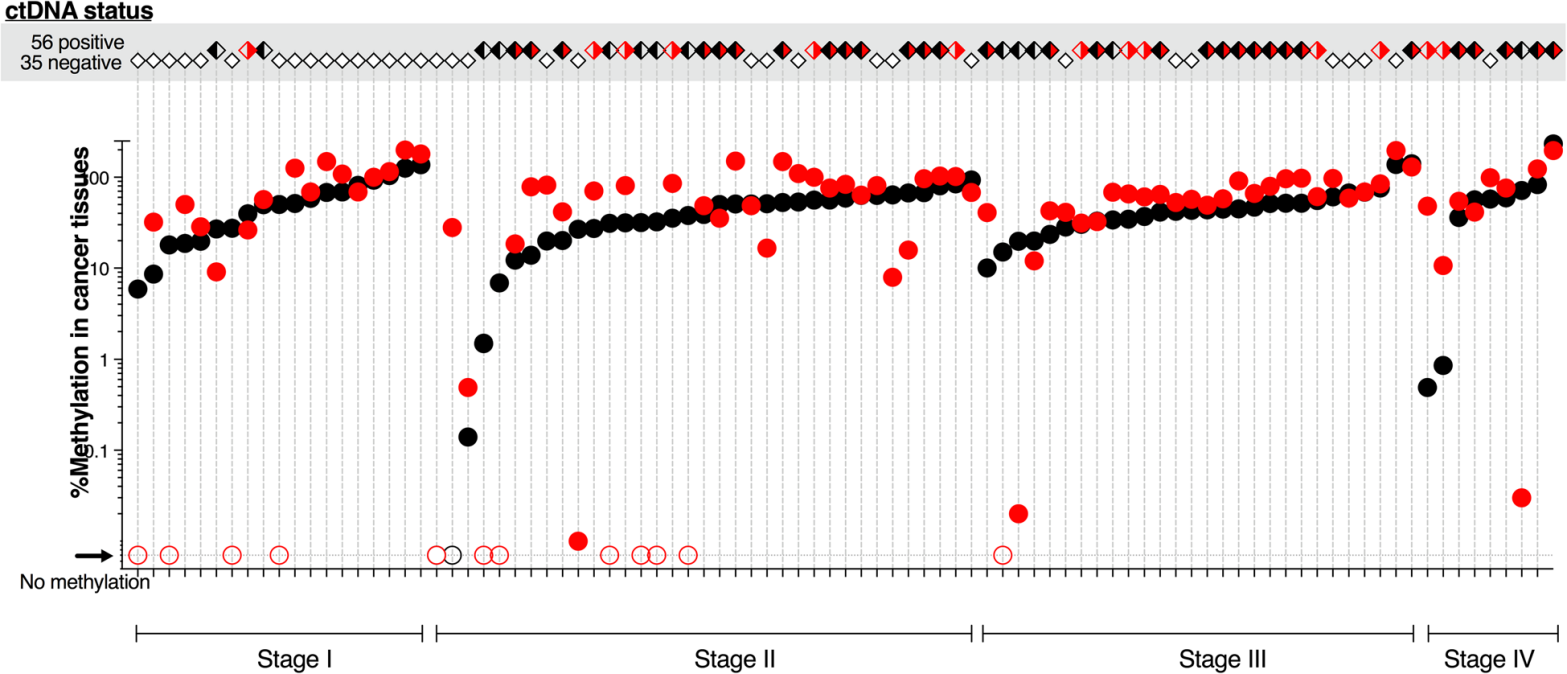
The case-by-case relationship between methylation in tissue and ctDNA positivity according to cancer stage. The top grey panel shows ctDNA positivity; open diamonds, ctDNA negative; black/white, *BCAT1* positive only; white/red, *IKZF1* positive only; and black/red, ctDNA methylated in both genes. The bottom panel shows graphical representation of methylation levels in cancer tissues (closed circles: black, *BCAT1*; red, *IKZF1*). Tissues with no detectable *BCAT1* and/or *IKZF1* are indicated with open circles

For the subgroup of patients who had surgical tissue assayed, there was no difference in methylation in tumour tissue between the 56 ctDNA-positive and 35 ctDNA-negative cases at peri-diagnosis (median (IQR): *BCAT1*, 43.4% (27.1–59.2) and 52.6% (26.1–68.2), respectively, *P* = 0.533; *IKZF1*, 59.3% (20.5–84.4) and 59.0% (16.6–100.3), respectively, *P* = 0.430). Detection of ctDNA was not concordant with tissue levels; levels of tissue methylation were not dependent on stage, whereas detection in blood was (Fig. 3). Most tumour tissues displayed elevated methylation of both *BCAT1* and *IKZF1* (*n* = 78, 85.7%), while ctDNA methylated in both genes was only detected in 30 patients (33.0%) (Additional file 1: Table S2).

### Post-surgery ctDNA status

**Fig. 4:**
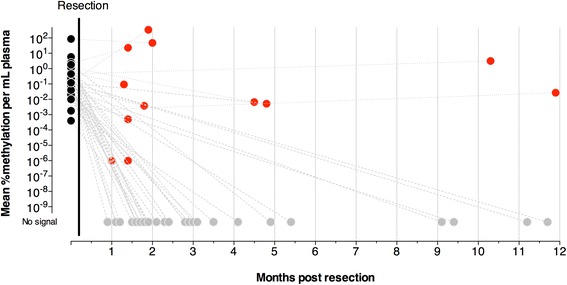
ctDNA status before and after resection of the primary cancer. The levels of ctDNA after surgery (within 12 months) are reported for the 47 cases who were positive for *BCAT1* and/or *IKZF1* ctDNA prior to surgical resection. Black circles, ctDNA positive cases before resection (*n* = 47); red circles, cases who tested ctDNA positive after resection (*n* = 12); grey circles, cases who tested ctDNA negative after resection (*n* = 35)

Plasma was available in 93 cases for detection of methylated ctDNA within 12 months of surgery (median (IQR) 1.9 months (1.6–4.0 months), Additional file 1: Figure S1). Thirty-five (74.5%) of the 47 cases who were ctDNA positive prior to surgery became negative after resection as shown in Fig. 4; plasma was collected in 26 of these within 3 months of surgery, indicating that reversion to a negative ctDNA status occurred rapidly. In the 12 cases who failed to revert to negative, eight had not received their full cancer treatment (i.e. adjuvant chemotherapy or resection of distant metastases) at the time of the blood collection.

## Discussion

This study explored how the methylated *BCAT1* and *IKZF1* DNA biomarkers for ctDNA related to cancer characteristics, aberrant methylation in CRC tissue and surgical resection as understanding these relationships might aid management of patients diagnosed with CRC by informing completeness of surgical resection.

For a ctDNA marker to be useful, the measured molecular features (e.g. mutation or methylation) should be present in the majority of cancer tissues and ideally should be independent of location, stage and molecular pathogenesis. Percentage methylation of either marker was much higher in cancer than non-neoplastic tissue, indicating that this was not a field effect. Our observations showed that all but one tumour had detectable methylation, with tumour tissue levels being independent of stage. The variables that related to degree of methylation in tissue was cancer compared to non-cancer tissue (either marker), age (lower methylation in younger people in cancer tissue, *BCAT1* only) and tumour location (higher methylation in proximal locations, *BCAT1* only). *IKZF1* also had slightly higher methylation levels in proximal cancers, but this did not reach statistical significance. Genome-wide hypermethylation has previously been reported to be more pronounced in proximal tumours than distal tumours [28]. Despite these differences in quantitative levels, there were no differences in the proportion of cancers considered positive for each methylated biomarker (i.e. with %methylation above the 75th percentile of non-neoplastic tissue). In addition, these differences were not reflected in the blood results, with distal tumours more likely to be ctDNA positive, which is likely to be related to morphological differences. Thus, *BCAT1* or *IKZF1* hypermethylation of tissue is common at all stages of CRC and appears to be more frequent than the somatic mutation frequency reported for known hot-spot genes such as *KRAS*, *TP53* and *APC* [5, 13].

ctDNA blood tests (regardless of whether the marker is genetic or epigenetic) appear to be limited in their capacity to detect stage I cases compared to all other stages [12, 29, 30]. There was only one patient who was negative for both methylated *BCAT1* and *IKZF1* in the tissue and who also returned a negative blood result. We report sensitivity for CRC of 62% across all stages, and the test was significantly more likely to be positive with more advanced clinical stage of cancer, which is consistent with our earlier findings [15, 16] and other commonly used CRC screening tests. In brief, we previously reported that at a matching specificity, the *BCAT1/IKZF1* blood test had equal sensitivity to a commonly used faecal immunochemical test (62 versus 64%, respectively) [16]. There are two commercially available molecular tests for CRC screening, ColoGuard (multi-target stool DNA test, including two methylation markers) and ProColon (methylated *SEPT9*). The multi-target stool DNA test has a reported sensitivity of 92% [31] but a lower specificity (87%) than what we report for the *BCAT1/IKZF1* blood test (92%) [16]. The estimated sensitivity and specificity for the ProColon blood test is 48 and 92%, respectively [30].

Our results show clearly that absence of *BCAT1* or *IKZF1* in blood at diagnosis does not reflect an absence of marker methylation in the tissue. Consequently, infrequent aberrant methylation is not likely to be a limiting factor for the *BCAT1/IKZF1* blood test. It is not clear if the usefulness of ctDNA testing for primary diagnosis is somehow biologically limited in early cancer and dependent on tissue vascularity, invasion or cellular turnover (e.g. apoptosis or necrosis). It is also uncertain if ctDNA biomarker assays are technologically limited in assay sensitivity, or if they will vary in usefulness between specific markers. However, it is likely that complex panels will be needed for ctDNA tests assaying for the less frequent somatic mutations, whereas ctDNA tests assaying for just a few methylation markers, such as those reported here, are sufficient. The literature is sparse on direct paired comparison studies (tissue versus blood), especially for methylation biomarkers. Using other molecular markers of cancer (microRNA, mRNA), Wang et al. reported the circulating levels of miR-601 and miR-760 to be significantly lower in CRC patients compared to that of healthy subjects, yet they found no differences in the expression levels of these miRNA markers between cancer and non-cancer tissues [32]. Conversely, good concordances have been reported for colorectal neoplastic tissue and plasma detection for CRC biomarkers such as *TYMS* and *LISCH7* mRNAs, [33, 34] *KRAS* mutation and *SEPT9* methylation, [35] albeit the levels in plasma were much lower than that found in tissue. For *Sept9*, methylation rates were 64.5% in tissue and 14.5% in plasma, while *KRAS* mutation was found in 33.6% of tissues and 2.9% of plasma samples [35].

There is considerable interest in understanding the association between ctDNA and detection of minimal residual disease and likelihood of overall recurrence and survival [9, 36]. In this respect, the epigenetic markers we applied showed that three quarters of patients who were ctDNA positive at diagnosis became negative after surgical resection. Many of the patients who remained positive had not completed their cancer treatment. The effect of surgical resection on detectable biomarker in blood adds credibility to the premise that these markers could be useful for monitoring of patients in that they are responsive to debulking. This observational study has been conducted in a usual-care moderate-sized clinical service where follow-up protocols are subject to variance according to practitioner protocols and risk for recurrence, rather than in the context of a formal highly structured prospective clinical trial where blood would be collected at set intervals following surgery and other therapies. Future prospective studies with tightly controlled and more frequent venipuncture would clarify the best time to draw blood after surgery, but it seems possible that it could be within 3 months, as most of the cases became negative within this time frame. A small study investigating the effect of surgery on mutation markers of ctDNA reported that cases became negative within 3 to 5 days [37]. All these observations are consistent with the estimated short half-life of circulating DNA [38] and indicate that these markers are highly and quite rapidly responsive to surgical debulking and thus adds credibility to the premise that these methylation markers are applicable for dynamic monitoring of residual disease [29]. As this study focused on the effects of surgery on ctDNA clearance, the length of follow-up was consequently not long enough to show what our findings mean for clinical outcomes such as risk for recurrence and death.

## Conclusions

Non-invasive analysis of ctDNA for post-treatment surveillance has the potential to become a practice-changing tool as it may create a window of opportunity for intervention at time points where curative modalities are still an option. Our findings demonstrate that detection of ctDNA using this noninvasive test for methylated *BCAT1/IKZF1* is informative with respect to completeness of surgical resection. Hypermethylation in the two investigated genes are near ubiquitous in CRC tissue, and their appearance in the blood as markers of ctDNA is related to cancer behavior (stage) and is not limited by lack of tissue expression. Testing for ctDNA by measuring for appearance of methylated *BCAT1/IKZF1* could aid patient management through selection of cases for intensified surveillance for recurrence and might allow identification of patients who would benefit from adjustments in adjuvant therapies.

## Additional files

Additional file 1: **Table S1.** Characteristics of patients included in analyses. **Table S2.** Concordance between detection of methylated *BCAT1* and *IKZF1* in tissues and blood. **Figure S1.** ctDNA status before and after surgical resection for 93 cases. (DOCX 36 kb)

## Abbreviations

*BCAT1*: Branched chain amino acid transaminase 1
CEA: Carcinoembryonic antigen
CRC: Colorectal cancer
ctDNA: Circulating tumour-derived DNA
*IKZF1*: IKAROS family zinc finger 1
