# Supplementary material for: Circulating tumour DNA for monitoring colorectal cancer—a prospective cohort study to assess relationship to tissue methylation, cancer characteristics and surgical resection
Source: Clin Epigenetics. 2018 May 16;10:63. doi: 10.1186/s13148-018-0500-5 (PMC5956533; doi:10.1186/s13148-018-0500-5)
Supplement: Supplementary file 1 — Table S1. Characteristics of patients included in analyses. Table S2. Concordance between detection of methylated BCAT1 and IKZF1 in tissues and blood. Figure S1. ctDNA status before and after surgical resection for 93 cases. (DOCX 36 kb) [file 13148_2018_500_MOESM1_ESM.docx]

**Supporting information**

**Additional file 1: Table S1. Characteristics of patients included in analyses**

| **Cases with:** | | **Peri-diagnostic ctDNA result** | **Available tissue** | **Post-surgery ctDNA result** |
| --- | --- | --- | --- | --- |
| Number of cases | | 187 | 91 | 93 |
| Age at diagnosis, years, mean (95%CI)^1^ | | 67.3 (65.6-69.0) | 67.8 (65.3-70.3) | 67.3 (64.9-69.7) |
| Gender, Male, No. (%) | | 112/187 (59.4) | 46/91 (50.6) | 48/93 (51.7) |
| Characteristics of primary cancer | | | | |
| Stage AJCC, No. (%) | Stage I | 40/187 (20.9) | 19/91 (20.9) | 30/93 (31.9) |
|  | Stage II | 54/187 (28.6) | 34/91 (37.4) | 30/93 (31.9) |
|  | Stage III | 63/187 (34.1) | 29/91 (31.9) | 31/93 (33) |
|  | Stage IV | 30/187 (16.5) | 9/91 (9.9) | 2/93 (2.2) |
| Location, No. (%) | Proximal colon | 75/187 (39.6) | 50/91 (55) | 44/93 (47.3) |
|  | Distal colon | 111/187 (59.4) | 41/91 (45.1) | 49/93 (52.8) |
|  | Unknown | 1/187 (0.5) | 0 (0.0) | 0 (0.0) |
| Size, mm (mean, 95%CI) | | 45.8 (42.8-48.8) | 46.6 (42.6-50.6) | 40.6 (36.8-44.5) |
| Lymphatic invasion, No. (%) | | 38/159 (24.2) | 25/91 (27.5) | 17/90 (18.7) |
| Perineural invasion, No. (%) | | 20/158 (13.2) | 13/91 (14.3) | 11/90 (12.1) |
| Poor differentiation, No. (%) | | 34/166 (20.9) | 26/86 (29.7) | 17/87 (19.8) |
|  | |  |  |  |
| ^1^ CI, confidence interval | | | | |

**Table S2: Concordance between detection of methylated *BCAT1* and *IKZF1* in tissues and blood.**

| Sample positivity | Cancer Tissue | | Blood | | Positivity, No. (%) |
| --- | --- | --- | --- | --- | --- |
|  | *BCAT1* | *IKZF1* | *BCAT1* | *IKZF1* |  |
| Positive tissue but negative blood | + | + | - | - | 29 (31.9) |
|  | - | + | - | - | 1 (1.1) |
|  | + | - | - | - | 4 (4.4) |
| Positive tissue and blood | + | + | + | + | 30 (33.0) |
|  | + | + | + | - | 6 (6.6) |
|  | + | + | - | + | 13 (14.3) |
|  | + | - | + | - | 7 (7.7) |
|  | + | - | - | + | 0 (-) |
|  | - | + | + | - | 0 (-) |
|  | - | + | - | + | 0 (-) |
| Negative tissue but positive blood | - | - | + | + | 0 (-) |
|  | - | - | - | + | 0 (-) |
|  | - | - | + | - | 0 (-) |
| Negative tissue and blood | - | - | - | - | 1 (1.1) |

**Figure S1. ctDNA status before and after surgical resection for 93 cases.**
